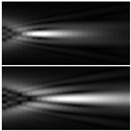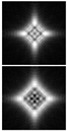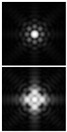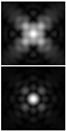# Modern Types of Axicons: New Functions and Applications

**DOI:** 10.3390/s21196690

**Published:** 2021-10-08

**Authors:** Svetlana N. Khonina, Nikolay L. Kazanskiy, Pavel A. Khorin, Muhammad A. Butt

**Affiliations:** 1 Image Processing Systems Institute of RAS—Branch of the FSRC “Crystallography and Photonics” RAS, 443001 Samara, Russia; khonina@ipsiras.ru (S.N.K.); kazanskiy@ipsiras.ru (N.L.K.); 2 Samara National Research University, 443086 Samara, Russia; paul.95.de@gmail.com; 3 Institute of Microelectronics and Optoelectronics, Warsaw University of Technology, Koszykowa 75, 00-662 Warszawa, Poland

**Keywords:** diffractive axicons, spatial light modulator, meta-axicons, nondiffracting beams, photonic integrated circuit based axicons, sensors based on axicons

## Abstract

Axicon is a versatile optical element for forming a zero-order Bessel beam, including high-power laser radiation schemes. Nevertheless, it has drawbacks such as the produced beam’s parameters being dependent on a particular element, the output beam’s intensity distribution being dependent on the quality of element manufacturing, and uneven axial intensity distribution. To address these issues, extensive research has been undertaken to develop nondiffracting beams using a variety of advanced techniques. We looked at four different and special approaches for creating nondiffracting beams in this article. Diffractive axicons, meta-axicons-flat optics, spatial light modulators, and photonic integrated circuit-based axicons are among these approaches. Lately, there has been noteworthy curiosity in reducing the thickness and weight of axicons by exploiting diffraction. Meta-axicons, which are ultrathin flat optical elements made up of metasurfaces built up of arrays of subwavelength optical antennas, are one way to address such needs. In addition, when compared to their traditional refractive and diffractive equivalents, meta-axicons have a number of distinguishing advantages, including aberration correction, active tunability, and semi-transparency. This paper is not intended to be a critique of any method. We have outlined the most recent advancements in this field and let readers determine which approach best meets their needs based on the ease of fabrication and utilization. Moreover, one section is devoted to applications of axicons utilized as sensors of optical properties of devices and elements as well as singular beams states and wavefront features.

## 1. Introduction

Mcleod was the first to coin the term “axicon” offers the most effective method for generating zero-order Bessel beams (hereafter represented as BBs), first published in 1954 [1]. There are two configurations of axicons: a positive axicon and a negative axicon. From the perspective of geometric optics, the positive axicon converges the incoming beams, while the negative axicon diverges the beams within a certain transmission distance [2,3]. For material processing, the created Bessel region is typically imaged onto the specimen to be processed by a lens setup involving two lenses or a lens and microscope objective. However, the Bessel region has a considerably increased peak strength relative to the original Gaussian beam (hereafter represented as GB) is still present immediately behind the axicon tip. High pulse intensities are known to cause plasma production and filamentation in the air, which should be avoided to ensure a safe and manageable ultra-short pulsed laser machining procedure. Negative axicons can be used to solve the generation of BBs with the additional focal length by splitting rather than superimposing the incoming GB [4]. A GB is transmitted through a negative axicon through a ring-shaped beam (later abbreviated as RSB) profile that can be focused later to create a BB. In comparison to the positive axicons, there is no additional Bessel region behind the optical element.

Laser beams in general have a Gaussian profile. An ellipsoidal focus formed by a lateral spot size and a confocal length is created when a GB is focused by an optical lens [5]. The congregating conical wavefront of an unbounded degree creates an ideal BB. The slanted conical wavefront makes the distinguishing BB shape as it assembles with the axis of symmetry and intermingles with itself. BBs are articulated in the far-field as a single ring pattern sprouting from its slender angular spectrum [6]. While a true BB would take a boundless aggregate of energy to be produced, an axicon delivers a decent estimation with virtually non-diffracting characteristics within the depth of focus (*DOF*) of the axicon [7,8].

The zero and high-order BB, also known as a nondiffracting beam (hereafter denoted as NDB), has several benefits, including preserving the size and shape of the central spot [9,10,11]. NDBs have been utilized in several applications, including laser machining [12], field depth extension [13], measuring multi-degree-of-freedom error motions [14], and 3D shape evaluation [15,16]. Vortex beams, specifically BBs, have recently attracted a lot of attention due to their unique properties in particle trapping [17,18,19], particle handling/rotation [20,21], and acoustic radiation force strategies in liquids [22]. BBs, known for their long depth of focus, have been used in material processing [23,24] and photopolymerization [25,26]. Their prospects for high-throughput 3D printing, on the other hand, have not been thoroughly examined. The BBs have a range of advantages, but they also have certain disadvantages. To begin, here are some energy characteristics: Due to the high energy cost, diffraction-free properties are achieved. Since only a small portion of the incident energy is concentrated in the central spot, the amount of energy on the optical axis decreases as the axial length of the beam increases. Furthermore, the presence of peripheral rings: as the focal spot shrinks, side lobes expand, worsening the image properties [27]. A detailed review of the applications of BBs can be found in [9,28,29].

**Figure 1:**
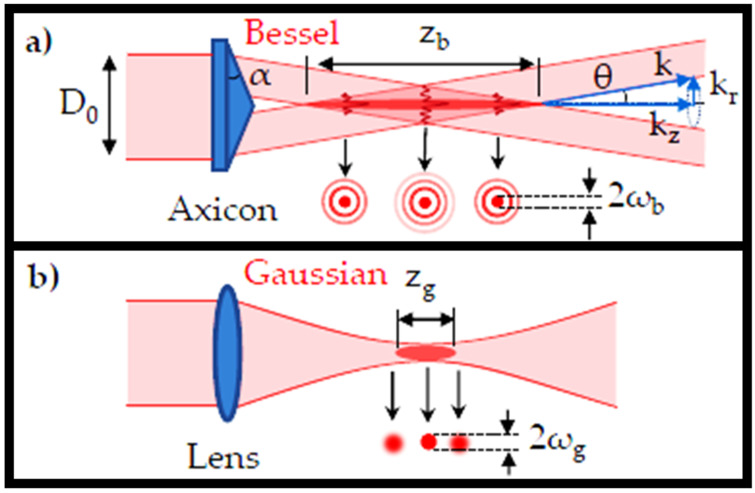
(**a**) Schematic of the BB generated by an axicon [12], and (**b**) the GB generated by a focusing lens [12].

As collimated GBs travel into an axicon with a refractive index *n* and a base angle *α*, they diverge from the optical axis by an angle of θ=arcsin(nsin(α))−α, as shown in Figure 1a [12]. The interference pattern generated by the conical crossing of wavefronts travelling through the axicon is known as the zeroth-order BB with the conical half-angle *θ*. The beam’s transverse profile consists of a sharp central core encircled by circular rings. The first zeros of the Bessel function will be used to calculate the maximum width/diameter of the central lobe (2*ω_b_*) [30].

$$2\omega_{b}=\frac{2\kappa }{k\sin\left(\theta \right)};\;\kappa \approx 2.405$$

The most intense part of the beam is the central lobe, which does not extend throughout the nondiffractive propagation (Bessel zone). The wavevectors of the BB propagating in a conical pattern can be used to estimate the length of the Bessel region *Z_b_* [31]:

$$Z_{b}=\frac{D_{o}}{2}\frac{k}{k_{r}}=\frac{D_{o}}{2\tan\left(\theta \right)}$$

where *D_o_* is the incident GB’s diameter. The energy density in the central lobe of any transverse plane within the Bessel zone would come from a consequent ring in the initial Gaussian allocation that is refracted by the axicon on this area. The energy (2ω*_b_/D_o_*)*E_in_* distributed over the surface area ($\pi \omega_{b}^{2}$) can be used to approximate the peak fluence of the central lobe (*F_b_*) [32].

$$F_{b}=\frac{2\omega_{b}}{D_{o}}\frac{2E_{in}}{\pi \omega_{b}^{2}}=\frac{4E_{in}}{\pi D_{o}\omega_{b}}$$

where *E_in_* denotes the Gaussian input energy. The half conical angle *θ* can be used to calculate the peak fluence.

$$F_{b}=\frac{2E_{in}}{\pi \omega_{b}Z_{b}\tan\left(\theta \right)}$$

The BB provides a considerably extended (typically one order of magnitude longer) depth of focus (DOF) than the Gaussian equivalent with a comparable beam diameter (2*ω_g_*~2*ω_b_*) than the normal GB, which is delivered by a focusing lens, as shown in Figure 1b [12]. The elongation of DOF (*z_g_* = 2 × Rayleigh range) in the Gaussian configuration will necessitate lenses with extended focal lengths and incident beams with narrow beam diameters. This traditional design has a wide range of applications and, unwantedly, produces a focused beam with a wide beam diameter, which limits uses for managing small features. In this case, the Bessel arrangement might provide a large DOF while keeping the beam diameter small.

An amazingly simple method was employed for the construction of the fluidic axicon [33]. As a model, a commercially available quartz axicon was used. This axicon was submerged in PDMS and treated. The quartz axicon was then separated, leaving a cone-shaped cavity inside the PDMS block. The block was then positioned between two glass slides, guaranteeing effortless and parallel optical faces. Through the PDMS, narrow inlet and outlet channels were created to permit the filling of the conical cavity with liquid of recognized refractive index [33]. Even though PDMS is utilized for the inverse molding of the axicon, it is feasible to use any polymer with high imitation fidelity and suitable optical characteristics. With a linearly polarized 1064 nm Gaussian beam, the fluidic axicon was illuminated. A charge-coupled device (CCD) camera was used to directly record IR images of the resulting BB. If a fluid with a refractive index higher than PDMS is utilized, a BB is formed instantly after the fluidic axicon. When the refractive index of the fluidic medium filling the void is less than that of PDMS, it is essential to add a telescope to create the BB. In [34], a liquid immersion axicon is produced that is capable of providing tunable BB, along with reducing the aberrations ensuing from the rounded tip of the axicon.

**Figure 2:**
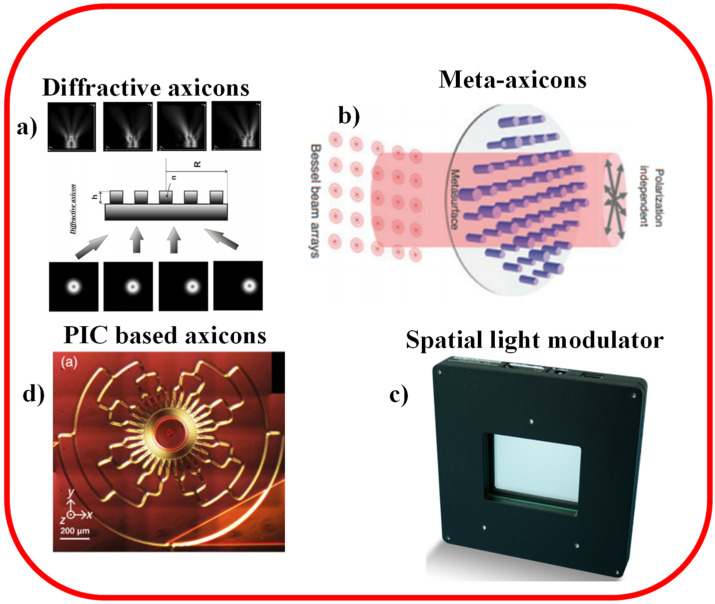
Methods to produce NDBs discussed in this paper, (**a**) diffractive axicon [49], (**b**) meta-axicon-flat optics [50], (**c**) spatial light modulator [51], and (**d**) PIC-based axicon [43].

Other methods have earlier been suggested for the expansion of tunable BBs comprising tunable acoustic gradients [35], lens imaging [36], and by means of the electro-optic effect in a uniaxial nonlinear crystal [37]. By spatial filtering in the focal plane of the primary lens utilized for imaging, aberrations arising from the rounded tips may also be reduced [38]. The fluidic axicons offer a compact, higher throughput and enhanced beam quality solution relative to these schemes. In this paper, we have reviewed the recent developments related to the methods used for the generation of NDBs by employing diffractive axicons [39,40], meta-axicons-flat optics [41], spatial light modulator [42], and photonic integrated circuits-based axicons [43,44], as shown in Figure 2. The range of NDBs produced by a real axicon is determined by the axicon’s parameters such as diameter and refractive angle [45,46]. When adjusting measuring ranges, it may be necessary to substitute the axicons with distinct angles or adjust the axicon’s cone angle [47]. It is inconvenient to substitute the axicon and modify the optical route and adjusting the cone angle is difficult [48]. The efficiency of NDBs and their applications would be affected by axicon fabrication errors. As a result, it is intriguing to offer a method for simulating axicons that can vigorously and compliantly modify the axicon’s parameters devoid of triggering fabricating errors. Our research group at Samara National Research University has been working on diffractive optical axicons for the last 10 years and we have successfully published several pioneering works in this field.

## 2. Refractive and Diffractive Axicons

When Durnin proposed the idea of NDBs, interest in axicons was reignited [52]. He showed a subset of optical fields that travel unaffected in free space since they are accurate solutions of the wave equation. The BB has an optical field whose amplitude is related to the zero-order Bessel function of the first kind, and it is an illustration of those fields that meet the axial symmetry criterion [52]. It has also been demonstrated that a BB can be created by combining a circular aperture and a regular lens, as seen in a typical axicon [53]. The optical field of an axicon-generated beam is only represented by a Bessel function in the near-axis approximation, making it a quasi-BB. However, because the optical field along the optical axis is important in a wide range of applications, BBs are still useful. Nondiffracting propagation and a micron-sized spot are two attractive properties of the BB. In laser machining, these beams have piqued scientists’ curiosity as an energy source [12,54]. Since BBs are nondiffracting, it is easy to demonstrate that they have many benefits over traditional beams when a BB shaped by a conical lens is employed instead of a traditional beam focused by a spherical lens. These benefits include: (1) a large gap between the workpiece and the mirror, which helps prevent ablated material from adhering to the lens, (2) high aspect ratio laser drilling is feasible, and (3) focusing does not necessitate precise alignment. In addition to these benefits, an axicon lens provides for a greater overall allowable misalignment than a standard lens [55]. Despite their poor energy efficiency (it is known that each ring and central spot of BB contains the same amount of energy [54], so the energy in the central spot is a small fraction of the entire beam), BBs have been proven to be encouraging for laser machining.

Laser beams with ring-shaped intensity allocations have drawn a lot of interest in science and technology for several applications. The utilization of optical elements for instance spiral phase plates [56] or axicons is popular in these applications for producing RSBs. However, since the functional numerical aperture (NA) of a RSB is smaller than that of a GB, the RSBs produced by these approaches have a non-diffraction-limited resolution. This is attributable to the propagating beam’s effective numerical aperture being reduced after passing through the element. The usage of a hollow optical fiber [57], a computer generated hologram (CGH) [58,59], and a liquid crystal cell [60] have all been documented as methods for generating RSBs. Recently, an axicon was proposed that would not require the use of an additional lens to perform the Fourier transform [61]. However, since the functional NA of a RSB is less significant than that of a GB, the RSBs in these applications have non-diffraction-limited precision. To solve the problem, a radial grating-based optical element is proposed to produce a diffraction-limited RSB [62]. The fine-tuning of the phase distribution in the RSB is an important part of maximizing the diffraction-limited RSB. The debris on a morphological structure manufactured by single-shot irradiation was significantly decreased in an experiment for fs-laser handling with a RSB because the resulting pulse did not disrupt the melt-solidification structure [62].

**Figure 3:**
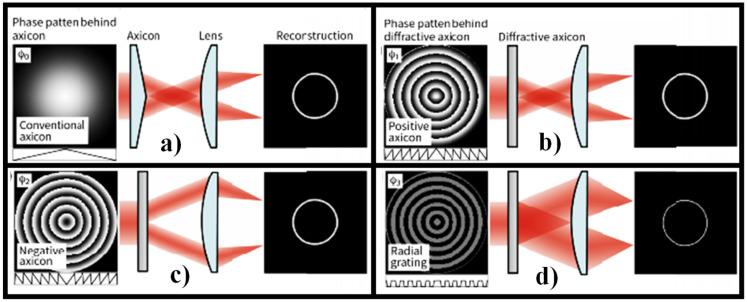
Phase patterns behind each axicon and their recreated forms [62], (**a**) standard axicon, (**b**) positive axicon [62], (**c**) negative axicon [62], and (**d**) radial grating [62].

Optical setups for generating a RSB are seen in Figure 3. To rebuild the beam, an axicon is typically used as seen in Figure 3a [62]. Figure 3b shows a diffractive axicon that is more compact. A BB with a longitudinal axial beam is also obtained between the diffractive axicon and a lens in this solution [62]. Since the lens may be affected by irradiation of the BB in the unlikely event of a high-power laser, the BB is unsuitable for some applications. Designing a negative diffractive axicon, as seen in Figure 3c, is one way to prevent such harm [62]. The lens is not damaged in this case; however, the RSB’s width is technically double that of the diffraction limit, since the maximum NA of the diffraction light is half that of standard focusing with a lens. For certain applications, the resulting reduction in resolution may be an issue. The setups in Figure 3a,b have the same problem. A radial grating is used to solve this problem, as shown in Figure 3d [62].

### 2.1. Refractive Axicons

The axicon was originally known in the form of a glass cone (refractive axicon). In classical optics, axicons are used to generate BBs [63]. Besides, convex or concave conical prisms (axicons) with Brewster angle can be used to generate nonuniform polarizations [64,65,66]. Downscaling the size of glass conic axicons to micrometer length scale via the expansion of advanced fabrication techniques significantly expands the application range of microaxicons or tapered fiber probes [67,68,69,70], allowing them to occupy an important place in micro- and nano-optics [71,72,73].

**Figure 4:**
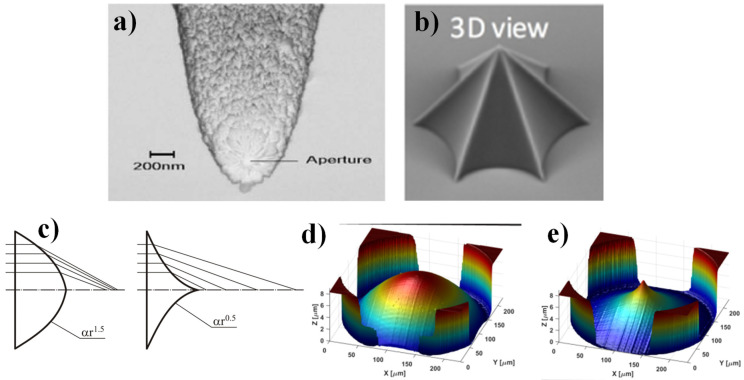
Nonconventional refractive axicons: (**a**) tapered fiber probe, (**b**) wrinkled axicon [86], and (**c**–**e**) fractional axicons [85].

Note that narrow axicons [73] with a small opening angle (about 20°, Figure 4a) are used as tapered fiber probes in scanning near-field optical microscopes (SNOMs) and can detect evanescent radiation [69,74,75], and also may be used as sensors of the longitudinal component of the electric field [76,77]. Lately, some adjustments of the classical conical axicon have been well thought out: gradient index axicons [78,79], axicons coupled with a spiral phase plate (SPP) [80,81] as well as logarithmic axicons [82,83], axicons with non-linear profiles [84,85] andwrinkled [86] (See Figure 4). Such structures, as a rule, combine several functions: not only the generation of Bessel-like beams, but also the introduction of a vortex phase, the formation of a uniform or spiral intensity, a decrease in the size of a light spot, and an increase in the resolution.

Refractive conic axicons have not only advantages such as high energy efficiency and low chromatic dispersion, but also disadvantages. Manufacturing of refractive axicons of anticipated quality poses some challenges due to the difficulty of technology and the lack of simple approaches of control and certification of conical surfaces [87,88,89,90].

In addition, refractive axicons have the numerical aperture (NA) limited by the angle of total internal reflection [73]:

$${\mathrm{NA}}_{tir}=\sin\left[\arccos\left(\frac{1}{n_{ax}}\right)\right],$$

where $n_{ax}$ is the refractive index of axicon’s material. For example, if $n_{ax}\;$= 1.5 (glass), then ${\mathrm{NA}}_{tir}\;$= 0.75. Higher NA values can be achieved using diffractive axicons, which can be used for sharp focusing and polarization conversions.

### 2.2. Diffractive Axicons

**Figure 5:**
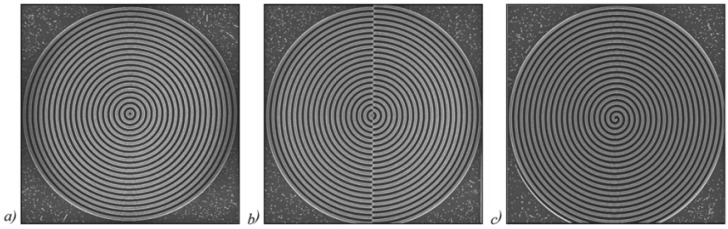
SEM images of different diffractive binary axicons: (**a**) axisymmetric, (**b**) bi-axicon, and (**c**) spiral axicon [96].

It is known that the axicon generates the zero-order BB with the central spot size at the half of the intensity maximum (full width at half maximum, FWHM) is 0.36 λ [91,92], which is 37% smaller than the size of the Airy disk (FWHM = 0.5 λ) formed by a lens with the same NA. This fact makes the axicon promising in applications where it is necessary to form a light spot compact in the transverse direction. However, for the linear polarization of the illuminating radiation, which is produced by most lasers, a decrease in the size of the focal spot in the total intensity of the electromagnetic field is prevented by the powerful contribution of the longitudinal component, which broadens the transverse size of the light spot along the polarization axis. In this regard, many studies have considered a radially polarized incident beam. With radial polarization, the high aperture axicon forms a light spot, consisting mainly of one longitudinal component, and makes it possible to overcome the diffraction limit predicted by the scalar theory in comparison with the lens [93,94]. Strengthening the longitudinal component is important in applications such as microscopy, high-resolution metrology, electron acceleration, and material processing [95]. To obtain a similar effect for uniformly polarized radiation (with linear or circular polarization) it has been suggested in the works [96,97] to use asymmetric diffractive axicons (See Figure 5). It is also possible to redistribute the longitudinal component to the central part of the focus due to the introduction of a linear or vortex phase singularity into the structure of the annular grating (or linear axicon) [98]. Axicons are also successfully used for efficient polarization and phase transformations in anisotropic media [99,100,101,102].

**Figure 6:**
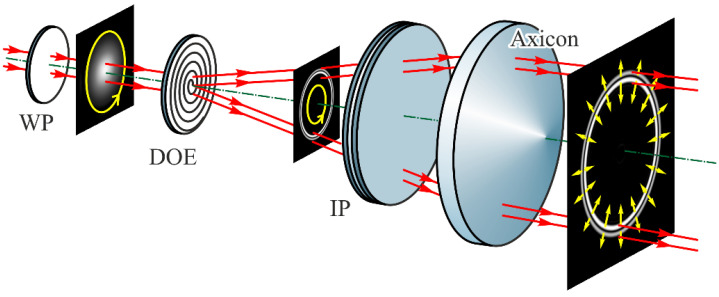
Optical scheme for converting a circularly polarized beam into a radially polarized vortex laser beam: a diffractive optical element (DOE) in the form of a binary phase axicon generates a conic wave with a defined divergence angle, an interference polarizer (IP) converts circular polarization into cylindrical polarization (radial or azimuthal in dependence of the conic divergence angle), and the refractive axicon (Axicon) transforms the conic wavefront into the plane wavefront [120].

Interesting and unusual properties are also provided by combining the two classic elements—the axicon and the lens. Typically, axicon-lens doublets (or lensacons) are investigated in the scalar paraxial approximation [103,104,105] and applied to vary the depth and lateral size of the focal area [106]. These properties of the lensacons are used in various optical systems [47,107,108,109] including medicine [110,111,112] as well as for laser structuring and micromachining [113,114,115,116,117]. In addition, lens-axicon combinations are used to control the polarization conversion of the beam in an anisotropic crystal [118,119] or multilayer anisotropic film (See Figure 6) [120].

**Figure 7:**
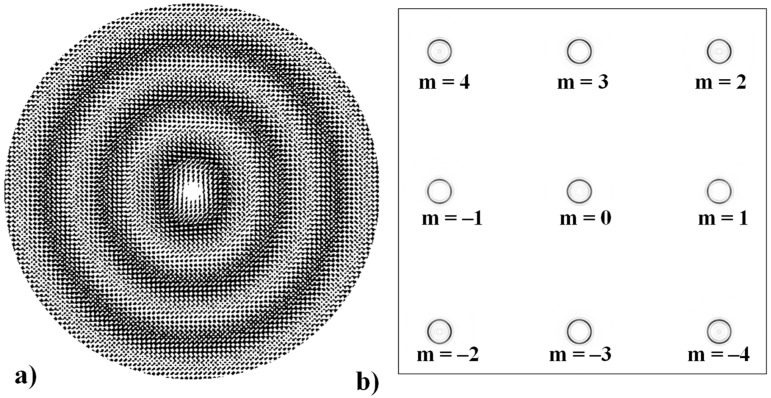
Simultaneous formation of several “perfect” optical vortices: (**a**) the phase of the 9-channel DOE, and (**b**) a set of focal rings with different vortex phases of the m^th^ order.

Axicon-lens doublets realize the Fourier transformation of BBs [121] are used to generate a light ring with the radius independent on vortex phase singularity (See Figure 7), named as the “perfect” optical vortices (POV) [122,123]. Such beams are used for optical capture and manipulation of microparticles [124], for free-space-optical communication [125,126], for high-resolution plasmonic structured illumination microscopy [127] in the study of noncollinear interaction of photons having orbital angular momentum (OAM) in the spontaneous parametric down-conversion process [128], as well as for the generation and detection of optical vortices outside the focal plane [129]. In the vector case, or when high-aperture optical elements are used (for example, a toroidal lens [130,131] may be used instead of an axicon-lens doublet), additional effects arise associated with both polarization transformations and redistribution of the 3D structure of the field intensity [132,133,134,135,136,137,138].

The use of spatial light modulators (SLMs) provides functions like those provided by DOEs. However, SLMs and DOEs are not interchangeable devices, as each has its advantages and disadvantages. SLMs tend to be supporting the implementation of a multi-level phase profile, while the fabrication of multi-level DOEs is not easy (just binary elements are the easiest to fabricate). The undoubted advantage of using SLMs is the implementation of dynamic control of the generated light fields. The limitations of SLMs are also well known: the relatively low damage threshold and efficiency of commercially available solutions, which somewhat limits the use of SLMs with high power lasers, for example, it requires additional SLM cooling systems [139]. DOEs makes it possible to obviate the challenges arising from the use of SLMs, namely their relatively low damage threshold and the need to use an additional optimization encoding for the realization of polarization changes [140,141]. However, SLMs are convenient dynamic devices of diffractive optics that are used in many optical applications.

## 3. Spatial Light Modulator (SLM)

**Figure 8:**
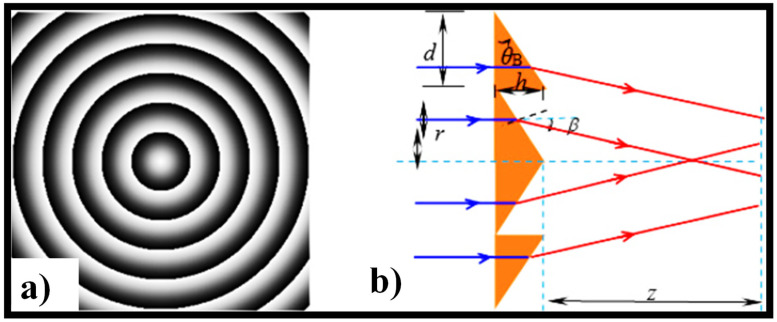
(**a**) A hologram for achieving the positive axicon [143], and (**b**) the central profile of the hologram [143].

Tunable axicons have been realized using several methods. The axilens, which incorporates the characteristics of an axicon and a spherical lens, was created using CGH optical components [103]. To obtain lensacons, a method established on a liquid crystal and phase-shifted electrical signals was suggested, with the logarithmic axicon as a model [48]. Some researchers used CGHs to create an arbitrary-order BB sequence that was nearly diffraction-free [58,142]. Since the perfect real axicon can produce nondiffracting BBs, fabrication errors can have an impact on the efficiency of NDBs. As a result, an easy-to-use approach is proposed. To attain phase modulation of the incident beam, the process involves loading the CGHs into the SLM. Figure 8a shows the hologram for mimicking the positive axicon, while Figure 8b shows the hologram’s central profile [143]. The profile can be seen as an arrangement of evenly spaced grooves, where d and h are the period and height of the grooves, respectively.

Pulsed ultrashort non-diffracting Gauss–Bessel beams (GBBs) may be beneficial in a range of applications, containing nonlinear optics and materials processing. SLMs can be employed as robust, extremely reconfigurable, vigorously manageable holograms, and their uses in the field of atom optics [144]. Light patterns that do not need microfabrication and can be projected into a vacuum system away from any surfaces can be created using such devices. A variety of light shapes that are challenging to create with traditional micro-optical procedures, but can be created with engraved holographic approaches, though they lack the versatility of the patterns created with the SLM. Though etched holograms can be used to create certain designs, the SLM has several advantages over these methods. The theories of diffraction, interference, and holography can be proven dynamically with real-time control over the constraints. Furthermore, SLMs make new studies feasible that are not practicable with traditional axicon model. For instance, SLM can also be employed to generate an array of BBs by adding the complex fields corresponding to an array of axicons and then extricating the phase of the resulting complex number as demonstrated in [145]. An array of 3 × 3 BBs is created and recorded on the CCD camera [145].

**Figure 9:**
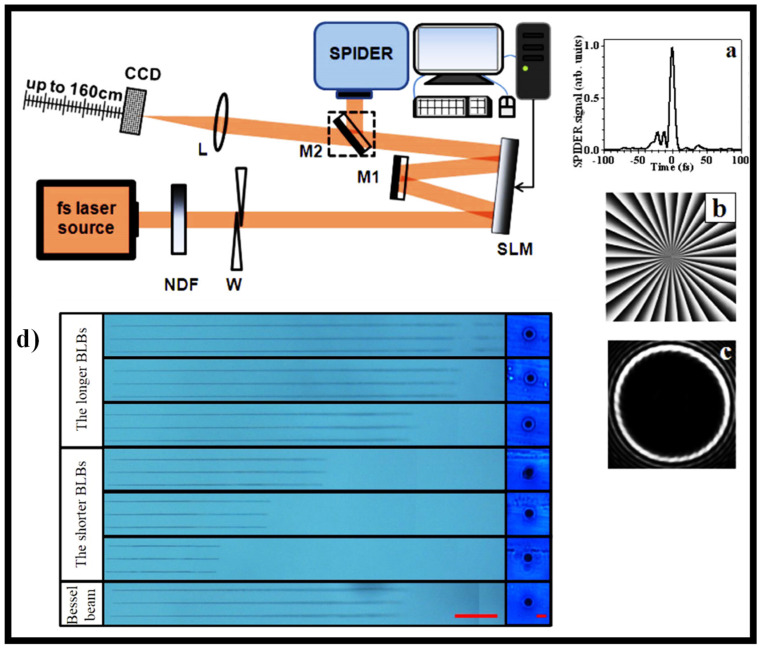
Experimental setup (on top) (**a**) measured time profile of the input fs-pulses entering the setup [146], (**b**) phase of an optical vortices [146], (**c**) typical RSB before Fourier conversion [146], and (**d**) drilling results for the three types of beams [the longer BLBs: R = +9 × 10^5^, +1.5 × 10^6^, and +5 × 10^6^; the shorter BLBs: R = −1 × 10^6^, −5 × 10^5^, and −3 × 10^5^; and BB [116].

Using a single reflective SLM, a simple but effective approach for producing zeroth- and first-order GBBs [146]. Diffraction half-angles of less than 40 μrad are obtained, and the propagation distance of the beam of more than 1.5 m is attained. In Figure 9, the experimental configuration is shown in the upper panel. To avoid damaging the SLM, the beam from a high-power few-cycle fs laser system is first controllably attenuated by a neutral density filter (NDF). The beam is then passed through a pair of fused silica wedges for accurate modification of the system’s minimum pulse duration. The pulses are characterized by spectral phase interferometry for direct electric-field reconstruction using a commercial device. Figure 9a shows an example of a normal pulse (duration 7.5 fs) [146]. Following the wedges, the beam enters the first half of the SLM, which has been programmed with the phase distribution of a highly charged optical vortex, as seen in Figure 9b [146]. The beam’s phase and amplitude are modulated, and the beam is diverted to M1, a clear silver mirror. The beam is reflected a second time at the other half of the correctly programmed SLM through this mirror. The topological charge of the highly charged optical vortices formed from the first reflection is reset to zero or one after this second reflection. Figure 9c depicts a standard RSB in the plane of the lens [146]. Due to their diffraction resistance, BBs are useful in high aspect-ratio micro-hole drilling. However, traditional BB generation approaches result in low adjustability of the nondiffraction length. The use of a phase-only SLM to generate Bessel-like beams (BLBs) with an arbitrary nondiffraction length is demonstrated [116]. Nondiffraction lengths ranging from 10 to 35 mm can be obtained using this process by altering the designed phase profile. The drilling results are shown in Figure 9d [116]. By spatially forming a fs laser beam, high-quality, high aspect ratio (560:1), and length-adjustable micro-holes can be drilled [116].

A phase transformation equivalent to the passage of the beam through an axicon is attained by the CGH displayed on the SLM [147]. The algorithm employed to generate CGH is simple and can be changed in real-time. Though, SLMs, have their downsides. SLMs are pricey and are susceptible to aberrations added in the production process, but it is possible to reduce the effect of these aberrations by introducing corrective terms to the CGH [148]. Recently, a low-cost SLM for practice in undergraduate and graduate optics labs is presented in [148]. For effective cone angles greater than several milliradians, due to the finite pixel resolution of the device, the accuracy of the BB may be compromised by aliasing. Furthermore, to displace the BB from the non-diffracted zero^th^-order spot, a blazing function must also be used. As a result, in comparison to a conventional axicon, a large amount of light is lost by the SLM. Usually, liquid crystal-based SLMs impose a strict constraint on the strength of the illuminating beam due to the possible harm incurred by heating, which may be a significant limitation for applications involving high laser powers. Lastly, the limited scale of numerous commercially available SLMs bounds the feasible transmission distance [149,150].

## 4. Meta-Axicons-Flat Optics

BBs are traditionally created using an objective with an annular aperture at the front focal plane, as suggested by Durnin [52,53], or with an axicon lens, as suggested by Herman and Wiggins [151]. The axicon lens solution was more commonly used since the first approach has poor quality, with most incident waves being obstructed by the aperture [129,152,153,154]. The introduction of metasurface (hereafter referred to as MS) flat optics [155,156], which allowed for advanced regulation of phase and amplitude on a subwavelength scale as well as management of dispersion properties, gave a new impetus to the expansion of axicon lens [157,158,159]. The Huygens theorem defines light propagation as a wavefront formed by the sum of spherical wavelets. Each meta-atom (MA) can be considered as the source of a spherical wavelet as light impinges on MSs. Surprisingly, the geometric variables, rather than the material conformation of nanostructures, govern the amplitude, phase, polarization, and even dispersion of spherical wavelets. The wavefront can be shaped at will in this way to produce flat optical elements with superior performance, even multifunctional ones, or to structure light by forming vector beams with complete control of polarization, which would otherwise need multiple optical components and be outside the competence of spatial light modulators (SLMs) [160]. This technique has been used to suggest and explain BB generators based on MS [161]. MSs are two-dimensional metamaterials made up of a group of subwavelength MAs that have been meticulously engineered [162,163,164]. By altering the shapes and orientations of the MAs on a subwavelength scale, these surfaces can have an impact on the phase, amplitude, and polarization of output light. MSs have recently attracted a lot of research attention because they provide a lot of versatility in terms of engineering their EM properties. MS’s special optical properties would allow for the implementation of a wide range of novel phenomena and functionalities not found in natural materials. MSs are now commonly used in nonlinear photonics, optical OAM [165,166], optical rotation [167], invisibility cloaking [168], metalenses [169,170], and holography [171,172], among other applications.

Most of these surfaces are made up of a collection of subwavelength MA arrays with precisely formed shapes and orientations [162]. As light propagates through an interface between two media, phase discontinuities are introduced. The Pancharatnam–Berry phase, also known as the geometric phase, is a common method for performing phase modulation using MAs with space-variant optical axis orientations [173,174]. Axicons integrated into optical fiber [175], cascading lenses [176], and metallic subwavelength MSs [177] have all been used to create BBs. Ultrathin subwavelength MSs have gathered a lot of coverage because of their outstanding benefits. MSs with spaced phase shifters have been used to monitor the optical wavefront and transmission of light, resulting in lenses, holograms, and polarization-selective devices that are all compact optical components. BB has been observed in both metallic and dielectric MS axicons [178,179]. MS-based devices may provide subwavelength spatial resolution, which is needed to deflect light by large angles contrasting to traditional phase modulating devices. This is required for high numerical aperture optical components, such as axicons and lenses, to produce beams with even smaller FWHM. Scanning microscopy [180,181], optical manipulation [182], and lithography [183], among other uses, all need subwavelength FWHM to attain high-level spatial resolution, strong trapping force, and subwavelength feature sizes, respectively.

The metallic elements, such as Ag and Au, were used in the groundbreaking work on MSs [184,185]. However, in the visible domain, polarization conversion limitations and internal ohmic losses of noble metals at optical frequencies have hampered the production of robust and cost-effective plasmonic-based MSs. Due to these limitations, researchers looked for suitable VIS-spectrum materials to create highly efficient MSs. Lossless dielectric materials with polarization-insensitive geometrics of their nano-resonators are the best candidate for addressing this problem, as they can ensure high efficiency for transmission-based geometries in the VIS-spectrum. Various research groups have shown exceptionally effective MSs using lossless dielectrics, such as TiO_2_, GaN, Si_3_N_4_, and others [179].

**Figure 10:**
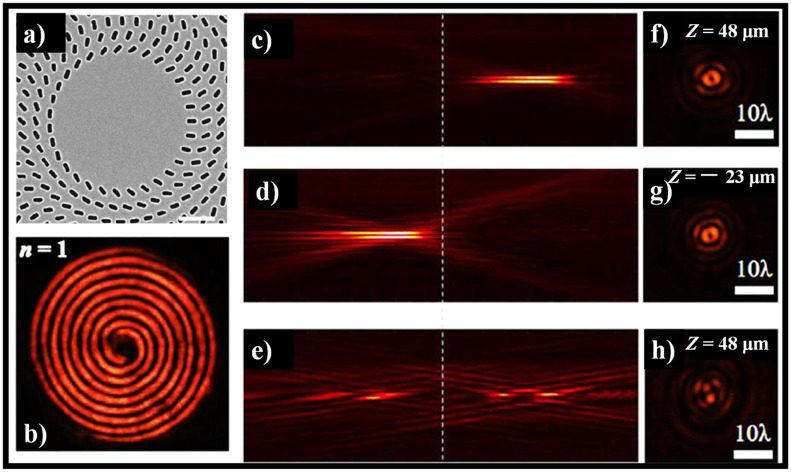
(**a**) SEM image of higher-order meta-axicons with topological charge, *n* = 1 [41], (**b**) interference pattern of high-order BBs [41], (**c**–**e**) intensity profile of higher-order BBs along the propagation direction at wavelength = 780 nm [41], and (**f**–**h**) corresponding cross-sections profiles [41]. The dotted lines stand for the sample plane.

One attractive possible application of MSs is the formulation of a meta-axicon based on these properties [186]. Recently, these properties were used to demonstrate a variable meta-axicon made up of rectangular nano-apertures assembled in numerous concentric rings that can focus left circularly polarized (LCP) light into a real BB and defocus right circularly polarized (RCP) light into a virtual beam [41]. By regulating the orientations of the nano-apertures, the desired phase discontinuity in cross-polarized transmitted light is added along with the interface. Furthermore, by properly designing the phase profile along the surface, meta-axicons can produce BBs of arbitrary orders. The meta-axicons have broadband optical properties, allowing them to change the wavelength of incident light from 690 nm to 1050 nm [41]. The SEM image of the meta-axicon is shown in Figure 10a. The intensity profiles along the propagating directions and cross-sections are shown in Figure 10b–h.

MSs are often used to implement polarization transformations, including the formation of cylindrical vector beams (CVBs) with radial or azimuthal polarization [187,188]. Among the various types of MSs, the most common are metal and dielectric subwavelength gratings [189,190], including nanostructured fused-silica q-plates [191,192] and S-waveplates [193] as well as structures of subwavelength anisotropic primitives (meta-atoms) [194,195,196]. Each type of MSs has its advantages and disadvantages. Metal subwavelength gratings work as a rule in a reflecting mode and are less chemically resistant to an aggressive medium, so all-dielectric MSs are preferable.

**Figure 11:**
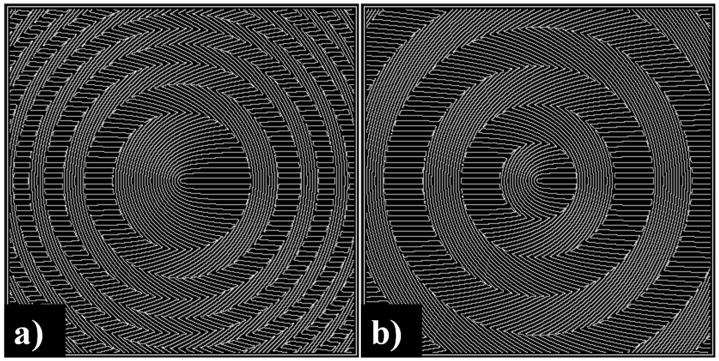
MSs for converting the linear polarization into the radial polarization and beam focusing: a combination of subwavelength polarization gratings with (**a**) zone plate (metalens) and (**b**) binary axicon (metaxicon).

The chief drawback of subwavelength gratings is the nonuniformity of the Fresnel reflection coefficient that arises because of the nonuniformity of a crystal’s refractive indices. However, this shortcoming can be evaded by joining subwavelength polarization gratings with a binary focusing element (See Figure 11), for example, zone plate or binary axicon [197,198,199,200]. In this case, the focusing element creates an additional phase, which is realized by rotations of the subwavelength grating grooves. A phase incursion of π radians corresponds to a rotation of the grooves by 90 degrees (Figure 11), and then all areas of the optical element will transmit radiation approximately uniformly.

## 5. Photonic Integrated Circuits Based Axicons

While most attempts to propagate NDBs are based on free-space optical applications [201,202], guided-wave optics is now searching for ways to ensure long-distance paralleled beam propagation. Self-collimation properties can be achieved using 2D photonic crystal (PhC) structures in the traditional technique [203,204,205]. However, it is mostly used to propagate single lobe GBs. At the subwavelength scale, the phase and amplitude of the wavefront must be adapted to implement a Bessel-type profile. It is difficult, if not impossible, to accomplish this mission utilizing a PhC solution established on the interference of waves travelling forward and backward in a slab waveguide (hereafter represented as WG) with a regularly altered dielectric index pattern. Integrating plasmonic nano-resonators on the surface of the dielectric WG, on the other hand, allows for efficient subwavelength modulation of the propagating wave’s phase and amplitude [206]. The fundamental method is established on the evanescent coupling of plasmonic nano-resonators to a portion of the light propagating in the WG. The local phase change of the directed wave is affected by the extra delay affected by the trapping of light by the nano-resonators. By tuning the geometric parameters of this hybrid MS-dielectric WG, such as evanescent field coupling in plasmonic nano-resonators, their surface density, resonance frequency, and quality factor, the modulation of the local phase and amplitude can be regulated. Effectual medium constraints for instance the n_eff_ of the WG can be added within the legitimacy limit of the homogenization technique, where all distinctive evaluations are far below the wavelength and the Bragg resonant interfaces can be ignored [206].

**Figure 12:**
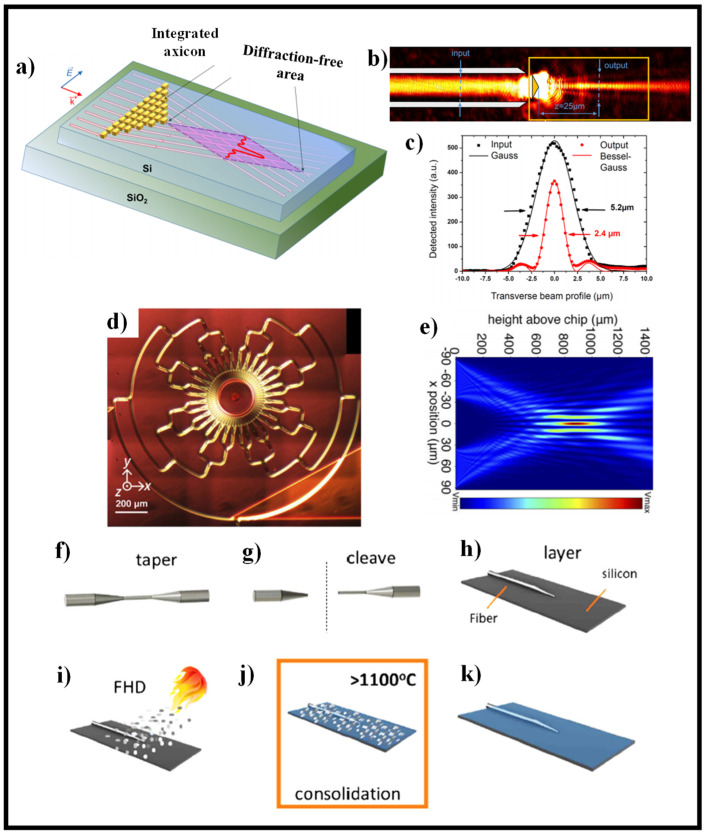
(**a**) Schematic of BB generator created by a 2D collection of plasmonic nano-resonators incorporated on an SOI WG [208], (**b**) SNOM view of the axicon [208], (**c**) cross-sections of the input and output beams with Gauss and Bessel-Gaussian fits, correspondingly [208], (**d**) microscope view of the manufactured device [43], and (**e**) modeling results utilizing two plane waves of sizes and field profile comparable to the chip-based evaluation to demonstrate an axicon [43]. Schematic illustrating consecutive steps (**f**–**k**) of integrated optical fiber construction and self-assembly of the axicon lens [175].

In [207], an MS-based axicon lens incorporated on a Si WG was used to produce near-field imaging of the propagating diffraction-free Bessel-type beam in a guided wave configuration as shown in Figure 12a. The axicon lens’ operation is focused on regional modification of the n_eff_ of the Si WG with plasmonic nano-resonators, which has a footprint of just 11 μm^2^. This generalized technique, which can be applied to a variety of planar lightwave circuit platforms, allows for the expansion of nano-engineered optical elements using plasmonic resonators to manipulate light at the nanoscale. The experimental characterization based on end-fire coupling and scanning near-field optical microscopy (SNOM) evaluations in collection mode is shown in Figure 12b [207]. Figure 12c gives the SNOM evaluation for the beam intensity profile before and after the axicon [207]. A photonic crystal-based device, in which the holes in the system were created using genetic algorithms, was another attempt to create integrated photonic lenses [208]. Although this unit has a beam waist of just 293 nm, the focus is only 5 μm from the device, and most notably, the axicon action is 2D, making it only suitable for light sheet applications. The small focus range, along with the reality that the focus is inside the slab itself, makes it unsuitable for imaging, optical traps, and other applications [208].

The on-chip axicon has previously been shown, but it is a 2D computer designed for light-sheet microscopy [209]. An axicon lens creates a plane illumination in light-sheet microscopy to minimize background noise when viewing a broad field of view. It needs lower excitation intensities in conjunction with fluorescence dyes than other fluorescence imaging techniques for identical image acquisition times and spatial resolutions. As a result, it is used in long-exposure experiments where a high dose of light may harm the sample. The silicon nitride-based devices direct light 10 μm from the chip’s edge into free space, with a beam waist of 1 μm [209]. Using optical phased arrays, another on-chip axicon-like system was exhibited in the Si_3_N_4_ substrate [210]. The system generates a quasi-1D BB of up to 14 mm Bessel length and an FWHM of 30 μm vertical to the direction of light propagation using a 1D splitter-tree based architecture. In [43], a NDB is produced using an on-chip SOI platform. Circular gratings with seven stages of 1 × 2 multimode interferometers make up the device. The authors present a method for azimuthally apodizing gratings by splitting the circles into arcs, which effectively improved the penetration depth in the gratings from ≈5 µm to ≈60 µm. The axicon, shown in Figure 12d is a 1.52 × 1.38 mm^2^ unit with 7 levels of 1 × 2 MMIs that merge, or split light and 128 final ports arranged around the central axicon structure [43]. Figure 12e depicts the simulation results of the E-field intensity distribution [43].

In [175], the self-assembly of an embedded micometer-scale oblate axicon refractive lens is reported. This falls under the category of “micro-axicon”, a subset of axicons that has sparked interest thanks to lens demonstrations on planar [211], fiber [212,213,214], and bulk materials [215]. The created lens resembles an oblate axicon, which when launched produces a quasi-BB that is guided in the substrate’s planar optical layer. The manufacturing process uses consolidated high-purity glass soot to monolithically mount an optical fiber to an optical planar substrate. The soot is deposited using flame hydrolysis deposition (FHD), which uses mass flow controllers to adjust the refractive index, stress, and thickness of the subsequent glass. The embedded optical fiber (IOF) network [216] has shown environmental reliability [217], physical monitoring [218], and refractometry [219] so far. Figure 12f–k demonstrates the fabrication process of IOF. For a detailed study, consult [175].

## 6. A Discussion of the Features of Various Types of Devices for the Implementation of Axicons

In this review, we examined four modern types of optical devices that allow implementing the functions of a classic conical axicon with additional options. In this section, we will briefly discuss the specifics of these implementations.

Refractive conic axicons have definite advantages such as high energy efficiency and low chromatic dispersion. However, manufacturing of refractive axicons of anticipated quality poses some challenges due to the difficulty of the technology and the lack of simple approaches of control and certification of conical surfaces. Another disadvantage is the limitation of the numerical aperture (NA) value by approximately 0.75.

Diffractive axicons are free from these imperfections, so they can be used for sharp focusing with NA ≈ 1 with sufficiently high energy efficiency. Moreover, diffractive axicons as annular gratings with a subwavelength period can be used for polarization conversions, which expands the functions of classical axicons. Diffractive optical elements (DOEs) are compact and relatively inexpensive optical elements, which can greatly simplify and reduce the size of optical systems. However, DOEs are characterized by a high chromatic dependence and provide a high-quality formation of a designed field only when illuminated by laser radiation with a certain wavelength matched to the height of the diffraction microrelief.

The use of spatial light modulators (SLMs) provides functions similar to those provided by DOEs. However, SLMs and DOEs are not interchangeable devices, as each has its features. SLMs tend to be supporting the implementation of a multi-level phase profile, while the fabrication of multi-level DOEs is not easy (just binary elements are the easiest to fabricate). The undoubted advantage of SLMs is the possibility of dynamic control of the generated light fields. The limitations of SLMs are also well known: the relatively low damage threshold and low efficiency of commercially available devices. This somewhat limits the use of SLMs with high power lasers, where it is safer to use DOEs. Moreover, SLMs are quite expensive devices. Thus, SLMs and DOEs with similar functionality are used in different applications depending on the characteristics of each device described above.

An interesting new type of axicons is meta-axicons. In addition to the compactness of such optical elements, the main advantage of metasurfaces is the ability to simultaneously perform both amplitude-phase and polarization transformations of the incident radiation. However, metasurfaces are more difficult to manufacture than diffractive gratings, since they have a substantially subwavelength characteristic structure size. Therefore, the most widespread metasurfaces have been acquired for the radiation of “long” wavelengths (THz and far infra-red range).

Note that such multifunctional compact structures as meta-axicons are very promising for use in waveguide structures. This is one of the rapidly developing areas, which requires a high technological base for the implementation/manufacture of various types of multiplexing and sensor devices.

In the next section, we examined the possibility of using simpler diffractive axicons to test the quality of optical devices, elements, and systems, as well as analyzers of wavefronts and laser beams.

## 7. Axicons Applied as Sensors

Bessel laser beams, which are formed by axicons, are very sensitive to wavefront distortions, which makes them useful for studying optical anisotropy and astigmatism of optical devices. A visually noticeable distortion of the beam intensity structure was observed for BBs when propagating perpendicular to the axis of an anisotropic crystal [6,220,221,222,223], as well as when passing through a cylindrical lens [223]. A similar transformation of the beam structure can be observed at an oblique incidence of a plane wave on an axicon [224,225,226]. In this case, changes in the intensity distribution for a BB are much more noticeable and occur at lower phase distortions than for Gaussian beams [225,227,228,229].

### 7.1. Optical System’s Astigmatism Detection

Let us consider the formation of a field by an astigmatic lens supplemented with an axicon. The transmission function of an astigmatic lens is as follows:

(1) $$\tau_{lens}\left(x,y\right)=\exp\left(-ik\frac{x^{2}+\mu y^{2}}{2f}\right)$$

where *μ* is the astigmatic coefficient, f is the focal length for rays in the XZ plane, $k=\frac{2\pi }{\lambda }$ is the wavenumber, λ is the radiation wavelength.

Let us supplement the lens (1) with a collecting axicon with the transmission function:

(2) $$\tau_{ax}\left(x,y\right)=\exp\left(-ik\alpha_{0}\sqrt{x^{2}+y^{2}}\right)=\exp\left(-ik\alpha_{0}r\right)$$

where $\alpha_{0}=\sin\beta$, *β* is the angle of inclination of the rays to the optical axis.

The field formed by the lens (1) together with the axicon (2) when illuminated by a plane wave in the paraxial approximation can be described by the Fresnel transform:

(3) $$G\left(u,v,z\right)=-\frac{ik}{2\pi z}\exp(ikz)\int_{-R}^{R}\int_{-R}^{R}\tau_{lens}\left(x,y\right)\tau_{ax}\left(x,y\right)\exp\left[\frac{ik}{2z}\left({\left(x-u\right)}^{2}+{\left(y-v\right)}^{2}\right)\right]d\;x\;d\;y$$

where *R* is the radius of the optical element, *z* is the distance from the element along the optical axis.

Equation (3) in the polar coordinate system takes the following form:

(4) $$\begin{array}{c} G\left(\rho ,\theta ,z\right)=-\frac{ik}{2\pi z}\exp(ikz)\exp\left(ik\frac{\rho^{2}}{2z}\right)\times \\ \int_{0}^{R}\exp(-ik\alpha_{0}r)\exp\left[ik\frac{r^{2}}{2}\left(\frac{1}{z}-\frac{1}{f}\right)\right]rdr\int_{0}^{2\pi }\exp\left(-ik\left(\mu -1\right)\frac{r^{2}\sin^{2}\varphi }{2f}\right)\exp\left(-\frac{ik}{z}\rho r\cos(\varphi -\theta )\right)d\varphi \end{array}$$

Obviously, if *μ* = 1 (there is no astigmatism), then the integral in Equation (4) over the angle is equal $2\pi J_{0}\left(\frac{k\rho r}{z}\right)$, and we obtain the well-known Fresnel transform for radially symmetric functions:

(5) $$G\left(\rho ,z\right)=-\frac{ik}{z}\exp(ikz)\exp\left(ik\frac{\rho^{2}}{2z}\right)\int_{0}^{R}\exp\left(-ik\alpha_{0}r\right)\exp\left[ik\frac{r^{2}}{2}\left(\frac{1}{z}-\frac{1}{f}\right)\right]J_{0}\left(\frac{k\rho r}{z}\right)rdr$$

when *z* = *f* (in the focal plane of the lens), Equation (5) corresponds to the Fourier–Bessel transform for a conical wave:

(6) $$G\left(\rho ,z=f\right)=-\frac{ik}{f}\exp(ikf)\exp\left(ik\frac{\rho^{2}}{2f}\right)\int_{0}^{R}\exp\left(-ik\alpha_{0}r\right)J_{0}\left(\frac{k\rho r}{f}\right)rdr$$

If the radius *R* is large enough, then the integral (6) with infinite limit (R→∞) can be calculated [230]:

(7) $$G\left(\rho ,z=f\right)=\frac{i\alpha_{0}}{kf}\exp(ikf)\exp\left(ik\frac{\rho^{2}}{2f}\right)\{\begin{array}{c} \frac{1}{{\left(\alpha_{0}^{2}-\rho^{2}/f^{2}\right)}^{3/2}},\;\rho <\alpha_{0}f,\\ \frac{-i}{{\left(\rho^{2}/f^{2}-\alpha_{0}^{2}\right)}^{3/2}},\;\rho >\alpha_{0}f. \end{array}$$

As follows from Equation (7), at $\rho =\rho_{r}=\alpha_{0}f$ there is a singularity, i.e., the field amplitude rises sharply. Thus, in the absence of astigmatism (*μ* = 1), a bright ring is formed in the focal plane.

If astigmatism is present, this will result in an angular intensity dependence. Field in Equation (4) then takes the following form [6,225]:

(8) $$G\left(\rho ,\theta ,z\right)\approx -\frac{ik}{z}\exp(ikz)\left[\Psi_{0}\left(\rho \right)+2i\Psi_{1}\left(\rho \right)\cos2\theta \right],$$

where functions $\Psi_{0}\left(\rho \right)$ and $\Psi_{1}\left(\rho \right)$ are defined by expressions:

(9) $$\begin{array}{c} \Psi_{0}\left(\rho \right)=\int_{0}^{R}\exp(-ik\alpha_{0}r)\exp\left[ik\left(-\frac{r^{2}}{2f}-\left(\mu -1\right)\frac{r^{2}}{4f}+\frac{r^{2}}{2z}\right)\right]J_{0}\left(\left(\mu -1\right)\frac{kr^{2}}{4f}\right)J_{0}\left(\frac{k}{z}\rho r\right)rdr,\;\Psi_{1}\left(\rho \right)\;\;\;\;\\ =-\int_{0}^{R}\exp(-ik\alpha_{0}r)\exp\left[ik\left(-\frac{r^{2}}{2f}-\left(\mu -1\right)\frac{r^{2}}{4f}+\frac{r^{2}}{2z}\right)\right]J_{1}\left(\left(\mu -1\right)\frac{kr^{2}}{4f}\right)J_{2}\left(\frac{k}{z}\rho r\right)rdr. \end{array}$$

**Figure 13:**
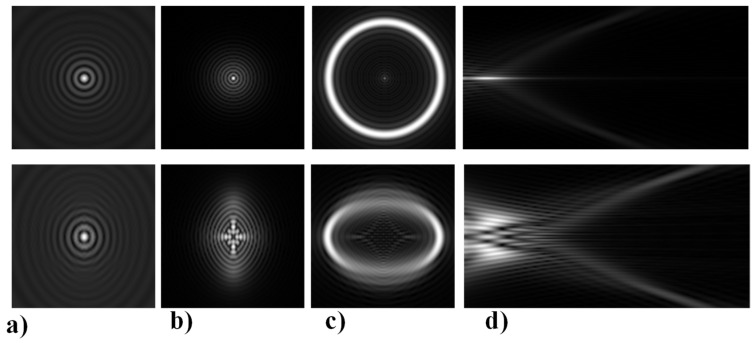
Simulation results for the plane wave diffraction on a lens supplemented with an axicon in the absence of astigmatism, *μ* = 1 (top line) and in the presence of astigmatism, *μ* = 1.1 (bottom line): amplitude distribution in the transverse planes (*u*, *v*∈[−2 mm, 2 mm]) at different distances from the lens: (**a**) *z* = 1500 mm, (**b**) *z* = 2000 mm, (**c**) *z* = *f* = 2500 mm, and (**d**) along the optical axis (*v*∈[−2 mm, 2 mm], *z*∈[2000 mm, 3000 mm]).

The results of modeling the diffraction of a plane wave on a lens supplemented with an axicon in the absence of astigmatism (*μ* = 1) and in the presence of astigmatism (*μ* = 1.1) are shown in Figure 13. In numerical modeling, the following parameters were used: λ = 633 nm, *R* = 10 mm, $\alpha_{0}=6$.33 × 10^−4^, *f* = 2500 mm. In this case, in the absence of astigmatism, a bright ring with the radius $\rho_{r}\approx 1.6\;\mathrm{mm}$ will be observed in the focal plane, while at astigmatism, the ring collapses into an ellipse (Figure 13c).

Note that the effect of astigmatism is visually more noticeable not in the focal plane, but at some distance to the focal plane at distance of *z* = 2000 mm from the lens (Figure 13b). In this case, for an ideal lens, a system of concentric rings characteristic of a BB is observed, and with astigmatism, the ring structure is noticeably distorted—a system of local maxima is formed in the central part.

As a rule, astigmatism of the focusing system is determined by the elongation of the focal spot, i.e., detection is carried out in the focal region. The proposed method with an additional axicon allows the detection of astigmatism in various areas—both in the focal area and at a considerable distance before and after (depending on the type of axicon) of the focal area.

Similar astigmatic transformations occur during the propagation of the BB in anisotropic crystals [6,220,221,222]. Therefore, the axicon can be used for mechanically free detecting birefringence of parabolic gradient-index lenses based on the astigmatic transformation of the zero-order BB [231]. Instead of concentric rings structure, the intensity of the BB acquires a diamond-shaped structure filled with local maxima. The number and intensity of these maxima are related to the degree of birefringence of the medium.

### 7.2. Measuring the Topological Charge of Vortex Beams

Vortex beams have a phase singularity of the form exp (imφ) and have the orbital angular momentum (OAM) equal to mħ per photon [232], where m is the topological charge (TC). Vortex beams are widely used in many fields of science and technology: laser manipulation of micro-objects, optical communications, super-resolution confocal optical microscopy, laser processing of materials, imaging optics and many others [233,234,235,236,237,238,239,240].

**Figure 14:**
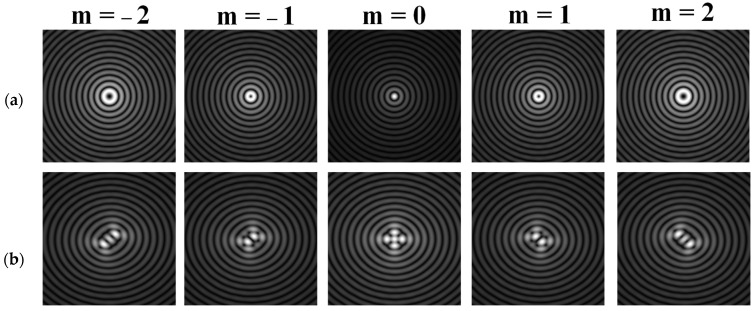
Determination of the value and sign of TC of BBs of various orders formed by the axicon: intensity patterns of (**a**) ideal beams (upper row) and (**b**) after astigmatic transformation (bottom row).

Various approaches have been proposed to detect and analyze the state of vortex beams, including those based on astigmatic transformation. Astigmatic transforms performed by a cylindrical lens [229,241], an inclined spherical lens [225,242], or biaxial crystals [6,222,243] enable to define the TC of a vortex beam. Additionally, astigmatic distortions in the structure of the BB occur when the radiation is obliquely incident on the axicon [224,225]. In the presence of a vortex phase singularity exp (imφ) in the illuminating beam, distorted patterns (a system of maxima in a diamond-shaped structure) will look different. This fact was used in [225] to determine the value and sign of TC of BBs of various orders (Figure 14). Ideal intensity distributions of BBs of various orders (when axicons illuminated by a normally incident beam) look like ring structures, from which it is impossible to determine TC (Figure 14, upper row). If we insert astigmatism, for example, by oblique illumination of axicons, then the pictures change significantly (Figure 14, bottom row). The TC |m| value can be determined by the difference in the number of maxima along different sides of the rhombus (Figure 14), and the TC sign—by the slope of the rhombus to the right or left.

Another approach based on the use of a displaced diffractive axicon was considered in [244]. In this case, the peripheral part of the axicon is used as a curved grating [245], which contains a cylindrical lens that performs astigmatic transformation and a linear grating that displaces the astigmatic transformed field outside the optical axis.

### 7.3. Wavefront Aberration Detection

Wavefront aberrations encountered in optical systems are usually described in terms of Zernike functions $Z_{nm}\left(r,\varphi \right)$ as follows [246,247]:

(10) W(r,φ)=exp[iψ(r,φ)],

(11) $$\psi \left(r,\varphi \right)=\overset{N}{\underset{n=0}{\sum }}\overset{n}{\underset{m=-n}{\sum }}c_{nm}Z_{nm}\left(r,\varphi \right)$$

where $c_{nm}$ are coefficients that determine the contribution of each Zernike function.

Various methods of wavefront detection are known, including interferometry [248], Shack–Hartmann sensors [249,250], multi-order diffractive optical elements that perform expansion in the basis of Zernike functions [251,252], as well as digital methods focused on phase recovery according to the intensity distribution patterns [253,254,255]. Moreover, for digital data processing, neural networks are increasingly used [256,257,258].

**Figure 15:**
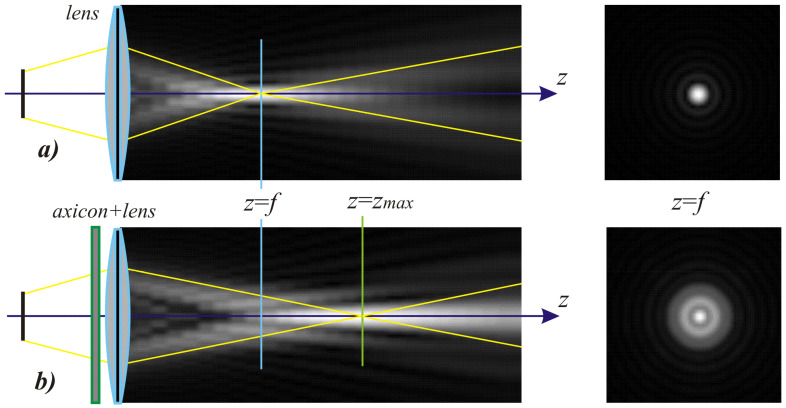
Optical scheme for (**a**) a single lens and for (**b**) a lens supplemented with an axicon: pictures of longitudinal amplitude distributions, as well as pictures of the amplitude in the focal plane (*z = f*).

It should be noted that at weak aberrations (small values of the coefficients in Equation (11)), the analysis of wavefront deviations from the focal pattern becomes difficult, since at weak aberrations the focal patterns differ slightly from each other, and in fact represent a bright focal spot characteristic to the ideal wavefront (Figure 15a). This problem is well known, and in this case the intensity in the extra focal planes is considered [255,257]. However, this requires moving the detection device, which is not always convenient or possible, especially for compact or stationary optical systems, for example, located on an aircraft or spacecraft board.

To solve this problem, we propose to supplement the focusing optical system with a diffractive axicon, as illustrated in Figure 15b. From a comparison of the longitudinal amplitude distributions for a single lens (Figure 15a) and for a lens supplemented with an axicon (Figure 15b), it can be seen that the presence of an axicon displaces the plane of maximum intensity along the optical axis. Note, when using a collecting axicon of the form (2), the displacement will occur closer to the input plane, and when using a scattering axicon $\exp\left(ik\alpha_{0}r\right)$, it will shift further from the input plane (as shown in Figure 15), and the displacement will be proportional to the axicon parameter $\alpha_{0}$.

Thus, when the lens is supplemented with an axicon, in the plane corresponding to the focal plane of the lens (*z = f*), instead of the focal picture, an out-of-focal picture is formed. This makes it possible to measure out-of-focal patterns without moving the detecting device. In addition, the depth of focus and its transverse scale increase, which are also positive factors for the analysis of images distorted by aberrations. The action of the proposed approach to improve the visualization of wavefront aberrations is shown in more detail in Table 1.

In conclusion, the analysis of the optical properties of devices, elements, and systems as well as features wave fronts and singular beams states can be detected and investigated using axicons which are standard low-cost optical elements.

## 8. Concluding Remarks

Several optical elements have been suggested in recent years for the effective production of non-diffracting beams (NDBs). These beams are widely used in several applications. Their transverse intensity profile’s propagation invariance can be used in metrology for scanning optical systems. Since they are less affected by atmospheric turbulence than other beams, they are therefore useful for large-scale evaluations. We have done our utmost in this paper to provide readers with up-to-date information on recent progresses in this fascinating area. For the generation of NDBs, we chose four fascinating devices: diffractive axicons, meta-axicons-flat optics, spatial light modulator, and photonic integrated circuit based axicons. When varying the measurement ranges, it might be important to use different refractive axicon lenses or change the axicon’s cone angle. Replacing the axicon and modifying the optical path is inconvenient and altering the cone angle is challenging. Axicon fabrication errors can reduce the performance of NDBs and their implementations. The issues concerning refractive axicon lens has been solved thanks to recent developments in this field. The use of spatial light modulators (SLMs) provides functions like those provided by DOEs. However, SLMs and DOEs are not interchangeable devices, as each has its advantages and disadvantages. SLMs tend to be supporting the implementation of a multi-level phase profile, while the fabrication of multi-level DOEs is not easy (just binary elements are the easiest to fabricate). The undoubted advantage of using SLMs is the implementation of dynamic control of the generated light fields. The introduction of metasurface flat optics, which allowed for advanced regulation of phase and amplitude on a subwavelength scale as well as management of dispersion properties, gave a new impetus to the expansion of axicon lens. The on-chip propagation of NDBs is an interesting way which has been recently investigated.

## Figures and Tables

**Table 1:** Simulation results for distribution near the focal plane in the presence of wavefront aberrations for a single lens (upper lines for each row) and a lens supplemented with an axicon (lower lines for each row).

Aberration Type	Longitudinal Amplitude Distribution	Transverse Distribution of Amplitude at Different Distances		
		*z* = 250 mm	*z* = *f* = 500 mm	*z* = 750 mm
Tilt 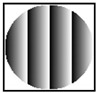 $Z_{1,1}\left(r,\varphi \right)=2r\cos(\varphi )$	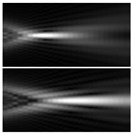	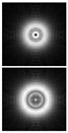	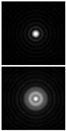	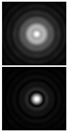
Defocus 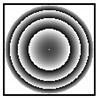 $Z_{2,0}\left(r,\varphi \right)=\sqrt{3}\;(2r^{2}-1)$	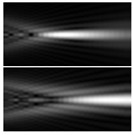	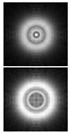	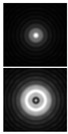	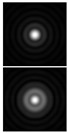
Astigmatism 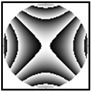 $Z_{2,2}\left(r,\varphi \right)=\sqrt{6}r^{2}\cos(2\varphi )$	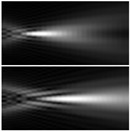	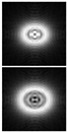	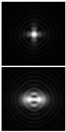	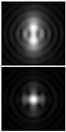
Pure coma 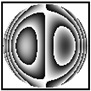 $Z_{3,1}\left(r,\varphi \right)=2\sqrt{2}\;(3r^{3}-2r)\;\cos(\varphi )$	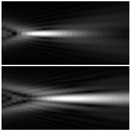	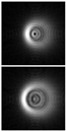	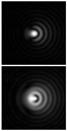	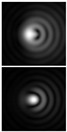
Trefoil 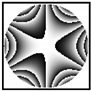 $Z_{3,3}\left(r,\varphi \right)=2\sqrt{2}r^{3}\cos(3\varphi )$	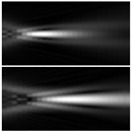	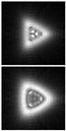	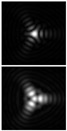	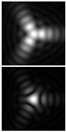
Quadrofoil 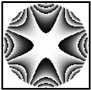 $Z_{4,4}\left(r,\varphi \right)=\sqrt{10}r^{4}\cos(4\varphi )$